# Antibacterial performance of nanocrystallined titania confined in mesoporous silica nanotubes

**DOI:** 10.1007/s10544-014-9847-3

**Published:** 2014-03-28

**Authors:** Krzysztof Cendrowski, Magdalena Peruzynska, Agata Markowska-Szczupak, Xuecheng Chen, Anna Wajda, Joanna Lapczuk, Mateusz Kurzawski, Ryszard J. Kalenczuk, Marek Drozdzik, Ewa Mijowska

**Affiliations:** 1 Centre of Knowledge Based Nanomaterials and Technologies, Institute of Chemical and Environment Engineering, West Pomeranian University of Technology Szczecin, Szczecin, Poland; 2 Department of Biotechnology, West Pomeranian University of Technology Szczecin, Szczecin, Poland; 3 Department of Pharmacology, Pomeranian Medical University, Szczecin, Poland

**Keywords:** Mesoporous silica nanotubes, Titanium dioxide, Antibacterial agent, Nanomaterials bactericidal properties

## Abstract

In this paper, we study synthesis and characteristics of mesoporous silica nanotubes modified by titanium dioxide, as well as their antimicrobial properties and influence on mitochondrial activity of mouse fibroblast L929. Nanocrystalized titania is confined in mesopores of silica nanotubes and its light activated antibacterial response is revealed. The analysis of the antibacterial effect on *Escherichia coli*. (ATCC 25922) shows strong enhancement during irradiation with the artificial visible and ultraviolet light in respect to the commercial catalyst and control sample free from the nanomaterials. In darkness, the mesoporous silica/titania nanostructures exhibited antibacterial activity dependent on the stirring speed of the suspension containing nanomaterials. Obtained micrograph proved internalization of the sample into the microorganism trough the cell membrane. The analysis of the mitochondrial activity and amount of lactate dehydrogenase released from mouse fibroblast cells L929 in the presence of the sample were determined with LDH and WST1 assays, respectively. The synthesized silica/titania antibacterial agent also exhibits pronounced photoinduced inactivation of the bacterial growth under the artificial visible and UV light irritation in respect to the commercial catalyst. Additionally, mesoporous silica/titania nanotubes were characterized in details by means of high resolution transmission electron microscopy (HR-TEM), XRD and BET Isotherm.

**Electronic supplementary material:**

The online version of this article (doi:10.1007/s10544-014-9847-3) contains supplementary material, which is available to authorized users.

## Introduction

During the past decades, scientists pay their attention to the environmental and health protection from the microbial hazard. One of the widely studied routs is photocatalytic degradation of the biological compounds. Since the titanium dioxide found application as a pigment and photoactive material detailed studies on its effective exploitation and modification are confirmed by numerous researchers (Alrousan et al. 2012; Awitor et al. 2008; Barreca et al. 2007; Buchel et al. 1998). Conventional methods of disinfection based on the ultraviolet light treatment require long time irradiation with high power ultraviolet lamp. More efficient method eliminating microbiological threats apply photoactive nanomaterials as catalyst (Butterfield et al. 1997), for water purification (Chen et al. 2011; Chen et al. 2008; Cendrowski et al. 2011) or antimicrobial films (Chong et al. 2010; Huguenin and Chopin 1998). Recently, many scientist focused attention to the synthesis, modification and application of titanium dioxide nanoparticles (Dai et al. 2011) and nanocomposites (Hashimoto et al. 2009; Jiang et al. 2009). According to the literature the enhancement of the photocatalytic performance is possible by: (i) modification titania nanoparticles with guest molecules like nitrogen (Kim et al. 2008a), (ii) metals and metals oxides (Kim et al. 2008b; Kmenta et al. 2010), (iii) synthesis of titania-graphene nanocomposites (Kumar et al. 2011; Mao et al. 2011) and (iv) deposition of titania particles on the surface of light weight templates with high surface area (Middlemas et al. 2013). The commonly studied compounds for synthesis of titania matrix are different nanostructured form of silica. Due to the easy syntheses and control over their shape, chemical and physical stability and low-toxicity. Additionally, their highly organized porosity, huge surface area and high pore volume make them perfect carriers for titania (Min et al. 2010; Mo et al. 2008).

From the current reports on the silica nanostructures synthesis, their various shapes are investigated e.g. solid silica nanospheres (Muruganandham and Swaminathan 2006; Obare et al. 2001), core-shell mesoporous silica nanospheres with hollow (Okamoto and Huang 2012) and solid core (Siwińska-Stefańska et al. 2012), different mesoporous hollow nanocapsules (Song et al. 2012), silica nanotubes with solid (Sui et al. 2013) or mesoporous walls (Tang et al. 2009). Studies on the synthesis and analysis of mesoporous silica nanotubes prove that these structures exhibit extremely high surface area, above 900 m^2^/g. Many scientist report novel and further modified routs of synthesis of silica nanotubes. One of the most common methods is the synthesis silica of nanotubes with carbon nanotubes as a tubular shape template (Wang and Song 2006). Similar methods use different nanorod-like metal structures as templates (Warheit and Toxicol. Lett 2013), but still carbon nanotubes are a frequently applied material. Their popularity derives from the extensive knowledge on their synthesis and vulnerability to high temperature, which enables easy removal of carbon template. Another technique is synthesis silica nanotubes inside anodic aluminium oxide (AAO) template. The advantage of synthesized nanotubes with the AAO template is a highly organised and uniform product (Wojtoniszak et al. 2010). Unfortunately, low efficiency of this methods is insufficient for any other application then laboratory study. Titania in anatase structure is a well-known photocatalyst, widely studied due to its availability, photoactivity and low cost (Wojtoniszak et al. 2012). Titania, at the same time acts as antimicrobial agent in presence of light (Wu et al. 2010).

This article describes a simple synthesis methodology of mesoporous silica nanostructers functionalized by TiO_2_ with enhanced bactericidal efficiency and photocatalytic properties. Furthermore, the correlation between cytotoxicity and the antibacterial activity of the samples has been revealed.

## Experimental

### Materials

Carbon nanotubes used to synthetize mesoporous shell was purchased from Shenzhen Nanotech Port Co. (Shenzhen, China). Silica (tetraethyl orthosilicate – TEOS) and titania precursors (Titanium(IV) butoxide - TBT) were purchased from Sigma-Aldrich. Ammonium solution, ethanol and n-propanol were provided by Chempure (Poland, Piekary Sląskie) and by Polskie Odczynniki Chemiczne - POCH S.A. (Poland, Gliwice). The commercial catalyst was provided by Evonic Industries, Aeroxide®P25 (TiO_2_-P25).

### Synthesis of mesoporous silica nanotubes with titania in the channels (CNT-mSiO_2_)

To fabricate a mesoporous silica layer, 0.2 g of multiwall carbon nanotubes were dispersed ultrasonically in a solution containing of 0.3 g hexadecyl(trimethyl)azanium bromide (CTAB – Sigma Aldrich), 0.67 ml of NH_3_ · H_2_O, 60 ml of ethanol (Chempure), and 80 ml of deionized water (H_2_O). The mixture was stirred for 18 h at room temperature after addition of 0.4 ml tetraethyl orthosilicate (TEOS – Sigma Aldrich) as the silica precursor. The suspension was centrifuged at 8,000 rpm for 20 min and thoroughly washed with ethanol. After removing the suspension residues, CTAB was burned out from the silica/carbon nanotubes in air at 400 °C for 3 h.

### Supporting of titania in the mesoporous silica nanotubes (tSiO_2_/TiO_2_)

The mesoporous silica/carbon nanotubes were sonicated for 3 h in 5 ml of concentrated tetrabutyl titanate (TBT – sigma Aldrich). Afterwards, the TBT and silica-carbon nanomaterials were diluted with propanol and collected after centrifugation at 8,000 rpm for 30 min. To remove excess of TBT, the sample was washed several times with propanol and then dispersed in ethanol to hydrolyse the titanium dioxide precursor. The mesoporous silica nanotubes with titania were obtained after calcination at 600 °C for 4 h to remove the CNT core and convert the titanium dioxide to the anatase phase.

### The antibacterial activity of the nanomaterials

The bactericidal effect of different silica/titania nanocomposites and commercial catalyst was examined by observation dayes in the numbers of the colony forming units (CFU) of *E. coli* (American Type Culture Collection - ATCC 25922). *E. coli* were cultivated in enrichment broth (Biocorp) for 24 h at 37 °C. Glass beakers of 0.1 L were used and 5 μL of the bacterial solution with density 3.01 × 106 /ml, was pipetted into the reactors and illuminated from above with UVA (4 × 20 W Philips) or Vis light (4 × 18W, Philips, TL-D 18W/33-640). Experiments without irradiation were also done. Nanomaterials/commercial catalyst (equal mass) were dispersed in the solution, in order to obtaine the concentration of 0.1 μg/ml. Dispersed nanocomposites with bacteria were stirred with the speed from 0 to 500 rpm. Control experiments were conducted in a distilled water with NaCl (0.9 %) added. All experiments were carried out at 37 °C for 45 min. Samples were taken every 15 min. Serial dilutions in NaCl solution (0.9 %) were prepared, and each of them was used to inoculate the bacteria on the Plate Count Agar (Biocorp, Poland). The plates were incubated for 24 h at 37 °C, and the colony-forming units (CFUs) were calculated. The experiments were verified three times.

### The *in vitro* cytotoxicity test

In sterile conditions, tSiO_2_/TiO_2_ were dissolved in phosphate buffered saline (PBS) and cell growth medium at six final concentrations (100 μg/ml, 50 μg/ml, 25 μg/ml, 12.5 μg/ml, 6.25 μg/ml, 3.125 μg/ml). The mouse fibroblast cell line (L929, ECACC) was cultured in 96-well culture plates (PAA) at an initial density of 1 × 10^4^ cells/well, and incubated at 37 °C in humidified 5 % CO_2_ atmosphere in 100 μl of culture medium [Dulbecco’s Modified Eagle Medium, High Glucose (DMEM, PAA, Austria) supplemented with 10 % heat-inactivated fetal bovine serum (FBS, Thermo Scientific), 0.4 % penicillin- streptomycin (Sigma) and L-glutamine (2 mM, Sigma)]. Following 24 h of incubation period, the culture medium was removed and suspensions of tSiO_2_-TiO_2_ were added. The activity of lactate dehydrogenase (LDH) in the medium was determined using a commercially available kit CytoTox96 Non-Radioactive Cytotoxicity Assay (Promega, WI, USA) according to the manufacturer’s instructions. The LDH leakage assay is based on the measurement of lactate dehydrogenase activity in the extracellular medium. The loss of intracellular LDH and its release into the culture medium is an indicator of irreversible cell death due to cell membrane damage. The untreated cells were used as a control (positive and negative). After 24 h, in order to measure maximum LDH release (positive control), 10 μL Lysis Solution was added and incubated for 45 min in a humidified chamber at 37 °C, 5 % CO_2_. After this time, the plate was centrifuged at 250×*g* for 4 min, in order to obtain a cell-free supernatant, and aliquots of the supernatant were then transferred into fresh 96-well flat-bottom plates. The reconstituted Substrate Mix (50 μl) was added to each well, and incubated at room temperature for 30 min, covered with foil for light protection. Finally, 50 μl Stop Solution was added to each well, and the absorbance was measured at 490 nm (with 620 nm background correction) using a spectrophotometric microplate reader (Sunrise Reader, Tecan, Switzerland). The readings were acquired from three independent experiments (each conducted in triplicate) using cells from different passages (2–10). Results were normalized to the control cells, and the percentage of necrotic cells was calculated using the following formula: % cytotoxicity = [(experimental LDH release – control / maximum LDH release – control)] × 100 %.

The influence of the nanostructure on mouse fibroblast cells mitochondrial activity, was assessed using WST-1 test (Roche Applied Science, Mannheim, Germany). The cell proliferation WST-1 test is based on the reduction of the tetrazolium salt WST-1 to a soluble red-colored formazan by mitochondrial dehydrogenases of metabolically active cells. The amount of formazan dye formed directly correlates with the number of metabolically active cells. For the present study mouse fibroblast cell line L929 was seeded into a 96-well plate at the density of 5 × 10^4^/well, and then cultured in a humidified incubator with 5 % CO_2_ at 37 °C. The cell culture medium (Dulbecco’s modified eagle’s medium D-MEM) was supplemented with 10 % of fetal bovine serum (FBS) and 1 % streptomycin/penicillin. After an incubation period of 24 h, L929 cells were exposed to the studied nanostructures in increasing concentrations, i.e. 0 and then 3.125; 6.25; 12.5; 25.0; 50.0; 100.0 μg/ml for 48 h. After the incubation period with the studied nanostructures, WST-1 reagent was added for 2 h, and the absorbance was measured at 450 nm wavelength using a micro plate spectrophotometer. The absorbance values were hence analyzed (calculating the average value from three wells per each experimental point in the case of the studied nanomaterials and from nine wells in the case of free medium PBS) to determine cell proliferation compared to control wells.

### Characterization techniques

HR-TEM micrographs were collected using an FEI Tecnai G^2^ F20 S Twin with an accelerating voltage of 200 kV and X-ray dispersion spectroscopy (EDX) was employed. Specific surface area of the samples was measured through N_2_ adsorption isotherm using the (interpreted with the Brunauer-Emmett-Teller or BET model) Quadrosorb SI (Quantachrome Instruments). Crystallographic phase identification was performed using X,Pert Philips PRO X-ray diffractometer (X,Pert PRO Philips diffractometer, CoKa radiation).

## Results

### Nanomaterials characterization

**Fig. 1:**
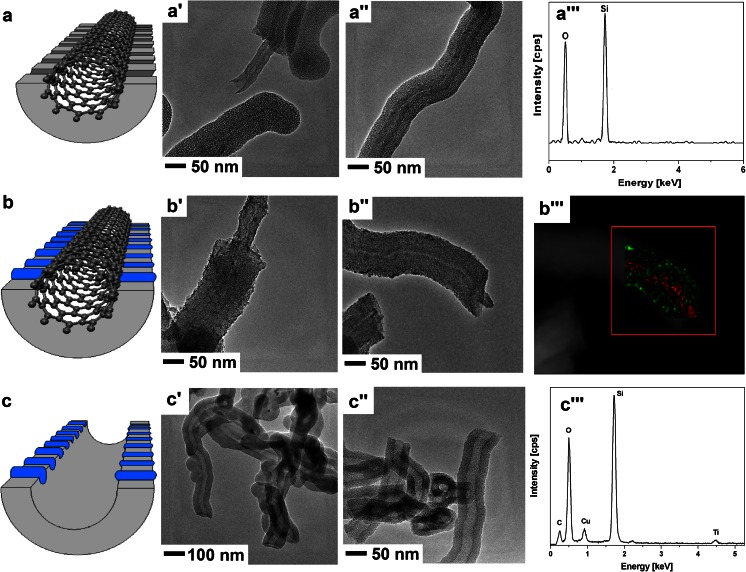
Draft and TEM images of mesoporous silica carbon nanotubes tSiO_2_/CNT (**a**, **a’**, **a”**), mesoporous silica carbon nanotubes supported with titania CNT-tSiO_2_-TiO_2_ (**b**, **b’**, **b”**), and mesoporous silica nanotubes with titania tSiO_2_/TiO_2_ (**c**, **c’**, **c”**). EDX spectrum of nanomaterials (**a”’**, **c”’**) and EDS mapping marked with *red colour* - carbon and *green colour* - titanium signal, proving the efficiency of titania modification (**b”’**)

In Fig. 1, the morphology of the mesoporous silica nanostructures before and after functionalization with the titania is demonstrated. The HR-TEM micrographs of silica/carbon mesoporous nanotubes clearly show that the surface of the multiwall carbon nanotubes is covered by the mesoporous silica shell with an average thickness of 30 nm (Fig. 1a’ and a”), and the elemental composition is determined by EDX (Fig. 1a”’). Both high-resolution TEM images in Fig. 1b’ and b” reveal the presence of an additional structure in the channels of the mesoporous silica after filling with titanium dioxide. In order to confirm the filling efficacy, EDX mapping was performed and the results are depicted in Fig. 1b”’. Here, the red colour corresponds to carbon and green stands for titanium. The inner space attributed to CNT is red and the outer space where the titania is supposed to be deposited is shown in green. The CNT-tSiO_2_-TiO_2_ is annealed in air at 600 °C to form anatase phase of titanium dioxide and remove carbon nanotubes. Figure 1c’ and c” clearly show the tubular structures without carbon nanotubes inside. However, the outer surface is unchanged with respect to CNT-tSiO_2_-TiO_2_. The efficiency of carbon nanotubes removal is confirmed by EDX (see Fig. 1c”’) which shows only Si, Ti, and O signals (Cu - copper signal comes from the TEM grid). The tiny signal of carbon in the spectrum may arise from amorphous carbon after burning.

For further verification of the morphological modification, the surface area is measured based on the N_2_ adsorption isotherm. The surface area of CNT-mSiO_2_ is 472 m^2^/g while tSiO_2_ exhibits surface area of 1,294 m^2^/g. It means that the specific surface area after removing carbon nanotubes increases by a factor of three. The specific surface area of the final sample, after filling the channels of mesoporous silica with titania and removal of carbon nanotubes (tSiO_2_/TiO_2_) dropped to 463 m^2^/g. Additional data with the N_2_ adsorption isotherms and the pore-diameter curve of the samples are given in Online Resource (Online Resource 1). The mean pore size diameter calculated based on the NLDFT method exhibits from 2.53 to 2.647 nm for tSiO_2_ and CNT-mSiO_2_ respectively. From the silica pore analysis, establish that its volume increases after the CNT thermal decomposition from 0.52 m^2^/g to 2.63 m^2^/g. After the silica pore fulfilling with titanium dioxide, pore volume decreases to 0.71 m^2^/g (volume calculated from the NLDFT method).

**Fig. 2:**
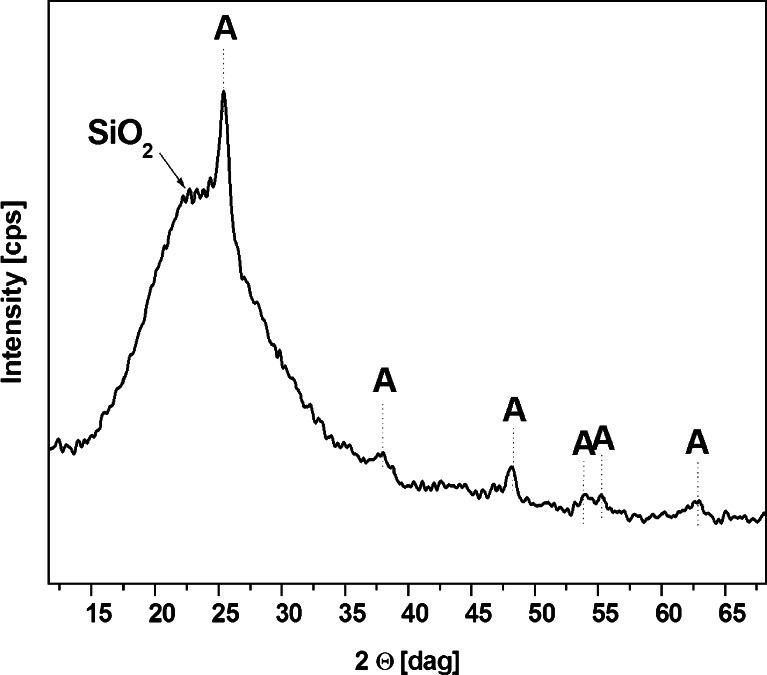
XRD patterns of tSiO_2_/TiO_2_ with marked main peaks from anatase and silica

Figure 2 shows the X-ray diffraction (XRD) patterns of tSiO_2_/TiO_2_ where the majority of the diffraction peaks can be indexed to the pure tetragonal anatase phase (ICDD #00-021-1272) of highly UV-light active titania. In the range of 15 to 30°, the broad peak corresponds to silica. The peak marked in the spectrum corroborate the high efficiency in the fabrication of mesoporous silica nanotubes with titanium dioxide.

### Biocompatibility study

**Fig. 3:**
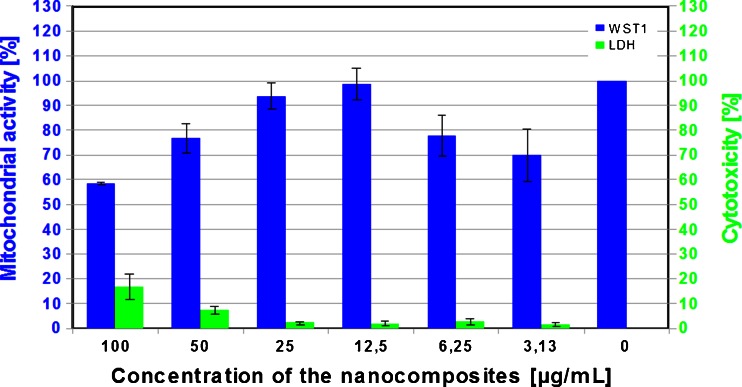
Mitochondrial activity (WST1 - *blue columns*) and cytotoxicity (LDH - *green columns*) to L929 mouse fibroblast of mesoporous silica nanotubes (tSiO_2_/TiO_2_) supported with the titania at the concentrations from 3.125 μg/mL to 100.0 μg/mL

Cytotoxicity potential of nanocomposites (tSiO_2_/TiO_2_) tested in L929 fibroblasts revealed their good cellular tolerance. Up to the concentration of 25 μg/mL, no marked toxic effects were found. Higher concentrations of the studied nanomaterials were characterised by slightly higher detrimental effects against the fibroblasts, but still even at the highest studied concentrations, the mean toxicity yielded only 16 % (Fig. 3 – green bar). According to the mitochondrial assays, mesoporous silica/titania nanotubes at highest concentration, decrease viability by about 40 % (Fig. 3 – blue bar).

### Antibacterial activity

**Fig. 4:**
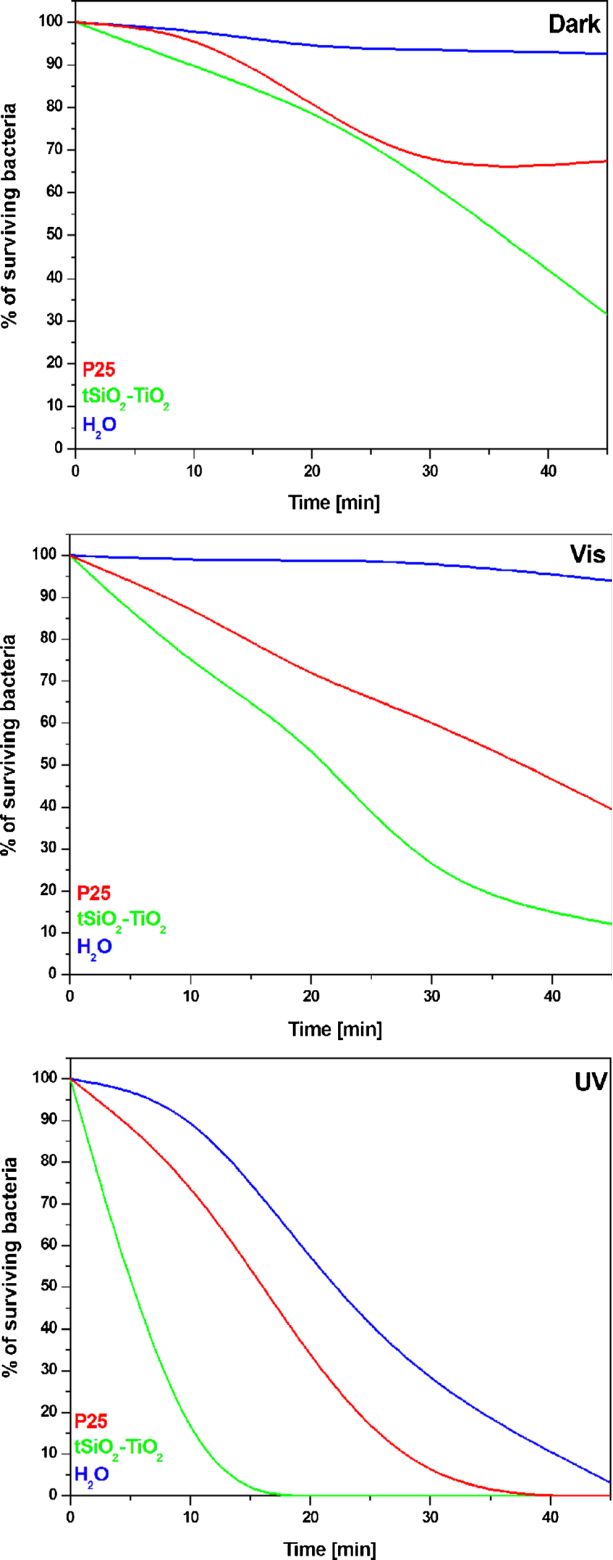
Diagram of photocatalytic-antibacterial performance (of mesoporous nanotubes tSiO_2_/TiO_2_) in darkness (*Dark*), under artificial visible light (*Vis*) and ultraviolet light (*UV*), compared to the performance of references sample: commercial catalyst (*P25*) and a blank comparator (*H*_*2*_ *O*), tested against *Escherichia coli* (ATCC 25922)

Results of the antibacterial activity of the tSiO_2_/TiO_2_ against *Escherichia coli* (shown in Fig. 4) clearly show effective growth inhibition compared to commercial TiO_2_-P25. The daily growth rates highly depend on the light conditions, and are strongly enhanced by the visible light. The mesoporous silica/tiatnia nanocomposites completely inhibit bacterial activity under UV light treatment and almost entirely kill bacteria under visible light (after 45 min irradiation). As a control the bacteria were exposed to the studied nanomaterial tSiO_2_/TiO_2_ and the commercial catalyst under non-light conditions (Fig. 4a). As an additional control, bacteria not exposed to antibacterial agents were cultured (marked H_2_O), to assess the self-disinfection effect. The water with addition of mineral components in order to reduce the osmotic stress (H_2_O), showed no significant inhibition of the bacterial growth. Both analysed catalysts show bactericidal activity without light activation. tSiO_2_/TiO_2_ catalyst inhibits bacterial growth stronger in comparison to the TiO_2_-P25.

The tSiO_2_/TiO_2_ nanocomposites exhibit bactericidal action under visible light irradiation at the level of 87 % of *E. coli* after 45 min (Fig. 4b). Commercial catalyst, TiO_2_-P25 shows enhanced antibacterial performance (at 60 %) in respect to the control (without photocatalyst) and under visible light conditions.

From Fig. 4c it proves that under UV light irradiation the tSiO_2_/TiO_2_ show further enhancement performance in the inactivation of *E. coli*. Almost 100 % of *E. coli* are killed in 15 min upon UV light irradiation. However while without the photocatalyst, 45 min is needed to kill *E. coli*. Figure 4a and b show bactericidal activity of tSiO_2_/TiO_2_ nanocomposites compared to the commercial catalyst under UV light and visible light irradiation, respectively. As shown in Fig. 4 under UV irradiation, commercial catalyst inactivates 100 % of *E. coli* after 45 min.

**Fig. 5:**
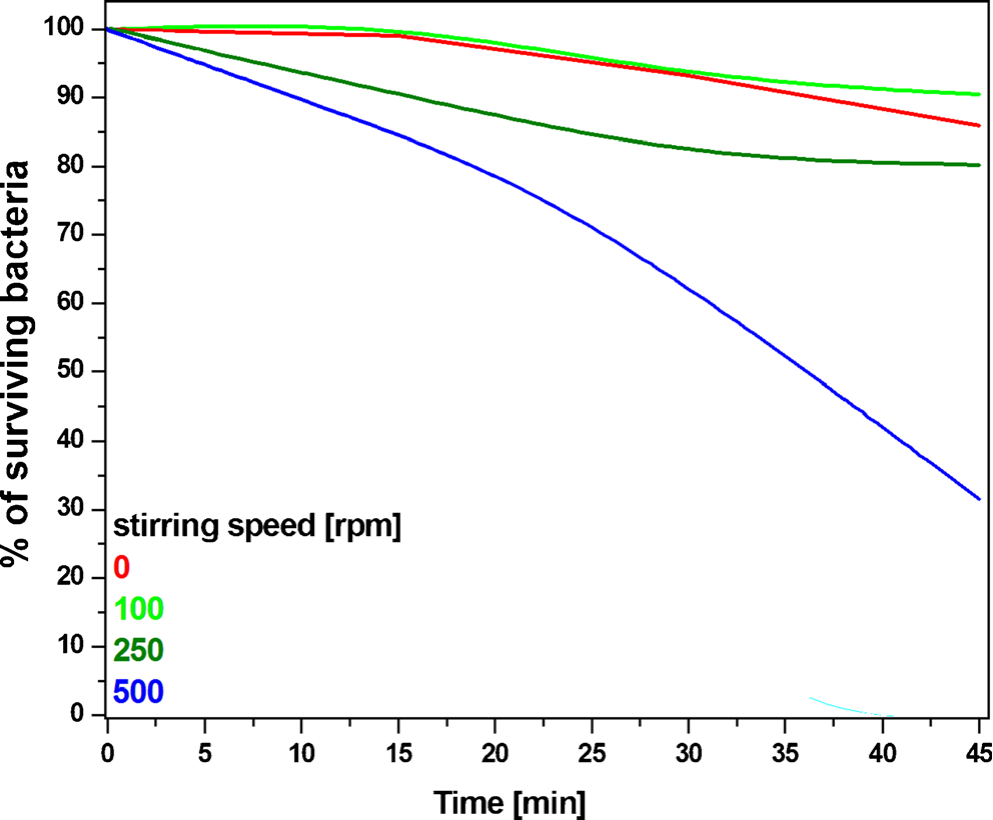
Diagram of antibacterial performance of mesoporous nanotubes tSiO_2_/TiO_2_ in darkness with different stirring speed of the solutions, tested on *Escherichia coli* (ATCC 25922)

In Fig. 4a, one can observe that the mesoporous titania silica nanocomposites show antibacterial activity even in darkness. In the studied nanomaterial, the inactivation of the bacteria is stronger with the reaction time, indicating that toxicity of the tSiO_2_/TiO_2_ plays an important but a limited role in killing *E. coli* during 45 min. In order to further investigate interaction between micro-organisms and nanocomposites, the bactericidal activity of the tSiO_2_/TiO_2_ was measured at different stirring rate (Fig. 5). The blank control experiments exhibit that cytotoxicity of the nanomaterials has low effect on bactericidal activity in the stationary conditions. Less than 15 % of E.coli are killed after 45 min. During the increase of the stirring speed up to 250 rpm killed 28 % of E.coli (after 45 min.) in the presence of the tSiO_2_/TiO_2_, while increase up to 500 rpm killed 70 % of the bacteria. Concentrations of all nanomaterials and bacteria used during the analysis of antibacterial activity were equal and prepared according to the section 2.4. All experiments carried out with the different light conditions (data presented in the Fig. 4) were conducted with stirring speed of 500 rpm.

**Fig. 6:**
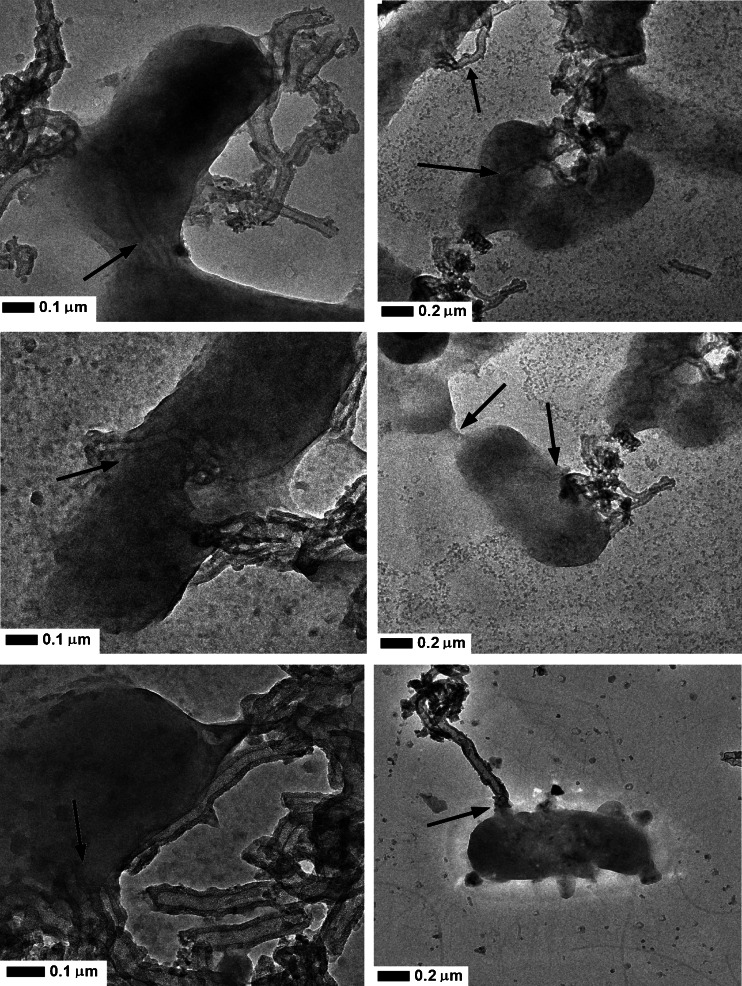
TEM images of the *Escherichia coli* bacteria exposed to mesoporous nanotubes tSiO_2_/TiO_2_, without irradiation (in *darkness*)

**Fig. 7:**
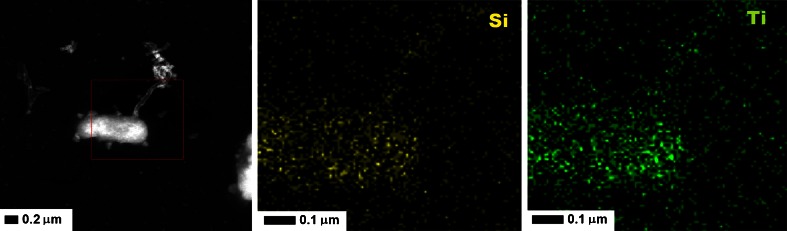
EDX mapping of the silicon (marked with *yellow colour*) and titanium (marked with *green colour*) elements of the Escherichia coli bacteria exposed to mesoporous nanotubes tSiO_2_/TiO_2_, without irradiation (in *darkness*)

Figure 6 shows the HR-TEM micrographs of titania confined in the silica mesoporous nanotubes, after 45 min. exposition to the E. coli bacteria (without stirring). The presented TEM micrographs clearly show that tSiO_2_-TiO_2_ were internalized by bacteria trough the membrane. Additionally EDX mapping was performed and the results are depicted in Fig. 7. Here, the green and yellow colour corresponds to titanium and silicon, respectively

## Discussion

In this contribution, nanocrystalline TiO_2_ successfully functionalized mesoporous silica nanotubes through a simple and efficient methodology. TiO_2_ supported in silica nanochannels was transformed into photoactive and crystallized TiO_2_ phase (anatase) through a high temperature treatment. The obtained nanomaterials also showed a high specific surface area due to the presence of hollow tubular mesoporous structure. This enables the tSiO_2_/TiO_2_ to inhibit bacteria growth more efficiently.

The bactericidal activity of the tSiO_2_/TiO_2_ nanocomposites, as compared to the control (without photocatalyst) and the commercial catalyst (P25), were studied against *E. coli* bacteria in dark conditions, and under artificial visible light and ultraviolet light irradiation. Both materials exhibited weak antibacterial activity in darkness in respect to the control sample without the catalyst. Nanocomposites tSiO_2_/TiO_2_ exhibit higher antibacterial activity then the commercial P25 in ultraviolet and visible light. The reason behind this is the light weight of silica template that significantly increased the amount of particles in the medium.

Figure 5 clearly indicates that the bactericidal effect of the inactivated photocatalyst depends on the stirring rate. The effect of antibacterial activity of the inactive photocatalyst, in static conditions (tSiO_2_/TiO_2_) corresponds to the toxicity of the nanocomposites and increases along with the stirring speed. According to Fig. 3, both mitochondrial activity and the amount of the released lactate dehydrogenase corresponded to the amount of living and dead cells respectively. Mesoporous silica/titania nanotubes exhibit low toxicity at the highest doses, at a concentration over 25 mg/ml. According to the data on the mitochondrial activity (Fig. 3 - blue bar), exposition of the cells to the nanomaterials reduced the activity. The difference between the released lactate dehydrogenase and mitochondrial activity suggest, that the nanomaterial interacts with living cells and inhibits growth of microorganisms. Similar correlation between the fibroblast cells and bacteria may indicate on bactericidal effect of the inactivated photocatalyst. In order to fully understand the interaction of the nanomaterials with the *E. coli*, the nanomaterial with bacteria was analysed in detailed by transmission electron microscopy (Fig. 6). Here it is clearly observed that *E. coli* adsorb mesoporous silica/titania nanotubes on its surface but also efficiently intake them through the cell membrane without significant changes in the bacterial structure (marked with the arrows in the Fig. 6). In order to confirm the assimilation of the silica/titania nanotubes inside the microorganism, elemental mapping by means of EDS was conducted (Fig. 7). The analysis has proved that bacteria partially assimilate silica and titania. As a result, the bacteria cellular proliferation can be disrupted. Wei Jiang et al. in (2009) made a similar observation in their study on the bactericidal effect of silica nanoparticles. There, titanium dioxide and other nanoparticles (ZnO, SiO_2_, Al_2_O_3_) exhibited signs of toxicity to the *E. coli* (Hashimoto et al. 2009). A detailed characteristic of the titanium dioxide and interaction of the *E. coli*, presented by Ashutosh Kumar et al. in (2011), suggests that after the internalization, nanoparticles induce oxidative stress resulting in DNA damage and cell death (Kmenta et al. 2010). Further microscopic analysis of the nanomaterial after bacterial exposition clearly showed that the structure of the photocatalyst was unaffected.

The photo-antibacterial activity of the tSiO_2_/TiO_2_ nanocomposites and commercial photocatalyst (P25) was evaluated under artificial visible light and ultraviolet light irradiation. The results of antibacterial properties of the samples performed under artificial visible light clearly indicate that the nanomaterial exhibits low antibacterial activity in comparison to the UV light irradiation, correlated to the partial range of ultraviolet wavelength of the emitted light. Even under partial UV light, anatase form of titania (active only in UV light) in tSiO_2_/TiO_2_ inhibited the bacterial growth by ca. 90 %. The commercial catalyst also exhibited antibacterial activity under artificial visible light, but with lower efficiency then mesoporous silica/titania nanotubes. According to King Lun (Yeung et al. 2009), microorganisms, due to heightened sensitivity, can be inactivated in the presence of weak UV light (Wu et al. 2011). Observation of Pinggui Wu et al. in (2010) shows that bactericidal activity of titania nanocomposites can be enhanced by visible light inducing oxidative lesions to the cell wall and cell membrane, further followed by the damage of interior DNA molecules, resulting in cells injuries and death (Wojtoniszak et al. 2012).

A considerable difference in the bactericidal activity was observed between tSiO_2_/TiO_2_ and the commercial catalyst. tSiO_2_/TiO_2_ required twice less time than the P25. Antibacterial photocatalytic effect of the commercial catalyst shows a slight improvement in respect to the antibacterial effect of ultraviolet light. The efficiency of antibacterial activity of the tSiO_2_/TiO_2_ irradiated by ultraviolet light is beyond the comparison with the self-bactericidal effect of nanocomposites without irradiation and with the artificial light. The direct synthesis of titanium dioxide inside mesoporous silica shell, having hollow, tubular shape, contributes to an increased surface area of the whole structure.

Future studies assume inner and outer body applications of the mesoporous silica/titania nanotubes, as a controlled antibacterial system. Low Visible and UV light, skin and organs penetration may be the main barrier of titania for the inner body application. Since titanium dioxide exhibits low UV light applicability for the inner body therapy, mesoporous silica/titania nanotubes show potential application as additives for skin surface protection (e.g. additives for bandages, gels). Promising studies have been reported on the application of silver nanoparticles (Zhenga et al. 2001; Amina et al. 2013) and nanocomposites of carbon nanotubes with polyvinylpyrrolidone-iodine (Anisha et al. 2013) as wound dressing additives . Studies on fabrication fibre modified with silver and titanium dioxide particles, reported by Yiyun Zhang et al. in (2013), show a potential application of the titania particles as bandage additives (Simmons et al. 2009).

Recent publications on titanium dioxide inner body applications focused on the titania X-ray activation (Schmidt-Stein et al. 2009). The main idea of the titanium dioxide inner body application is its photooxidizing effect, based on the production of free radicals, resulting in the degradation of carbon-spices (Liu et al. 2009), DNA damage and cell death (Kmenta et al. 2010). Due to the control over the time and power of titanium dioxide irradiation (UV/X-ray), we receive a control over its therapeutic effect. During the activity analysis of a potential photo-antibacterial agent, it is important to focus only on the bactericidal performance induced with the UV/Vis light irradiation. Due to the fact that nanomaterials may simultaneously exhibit cytotoxicity effect and bactericidal properties (caused by the oxidation stress triggered by the titanium dioxide irradiation), biocompatibility of the tSiO_2_/TiO_2_ is a highly important factor. The silica and titanium dioxide nanotubes biocompatibility, proved with the mitochondrial and lactate dehydrogenase activity assay, allows to distinguish the cytotoxicity effect of the nanostructure from their bactericidal performance.

Many available data confirm high biocompatibility of silica nanotubes with human cells, especially at lower concentrations (0.05–0.005 μg/ml) (Dantigny et al. 2005). Likewise, TiO_2_ alone has no effect on cell proliferation, but it becomes cytotoxic after UV irradiation (Nan et al. 2008; Kubota et al. 1994). Following UV excitation, activated TiO_2_ nanoparticles demonstrate oxidation and reduction activity, and produce various chemical changes (Wang et al. 2011). Wang et al. showed that photoexcited TiO_2_ exert potent cyto- and genotoxic effects and can induce cells apoptosis (Cai et al. 1992). Therefore, SiO_2_ containing TiO_2_ nanoparticles could be of potential use in the photodynamic therapy—e.g. cancer therapy (Wang et al. 2007). Furthermore, TiO_2_ nanotubes are promising carriers that deliver drug to target sites, and release them upon different stimuli such as UV light, pH, temperature (Yamaguchi et al. 2010).

In the future, further research is planned on the antibacterial activity of the titanium dioxide (confined in the mesoporous silica nanotubes) with the X-ray as an activation source and enlargement of the bacteria test group (the gram-negative bacteria—eg. Shigella dysenteriae, E coli—research continuation; the gram-positive bacteria—eg. *Staphyloccocus aureus*, *Streptococcus pyogenes*).

## Conclusion

In conclusion, mesoporous silica nanotubes, modified with nanocrystilline titanium dioxide (anatase phase) placed in the pore, were successfully fabricated and used as a photoactivated antibacterial agent. The photocatalyst shows more efficient bactericidal properties compared to the controls (water, P25) under artificial visible and ultraviolet light. tSiO_2_/TiO_2_ nanocomposite exhibits similar toxicity in both examined cells L929 mouse fibroblast cells and Escherichia coli. The study revealed a strong correlation between the stirring speed of the tSiO_2_/TiO_2_ and the toxic effect of the nanomaterial on microorganisms. The conducted analysis of the bactericidal effect proves the intake and internalization of the tSiO_2_/TiO_2_ through the cell membrane of the microorganism, and its influence on the cells proliferation. The comparison of the anti-bacterial and photocatalytic efficiency of the tSiO_2_/TiO_2_ clearly indicates that nanotubes exhibit low toxicity and can be proposed as a future generation antimicrobial and cleaning system for the environmental and health protection.

## Electronic supplementary material

Below is the link to the electronic supplementary material.

ESM 1 (DOC 523 kb)
