# Supplementary material for: Antibacterial performance of nanocrystallined titania confined in mesoporous silica nanotubes
Source: Biomed Microdevices. 2014 Mar 28;16(3):449–58. doi: 10.1007/s10544-014-9847-3 (PMC4009144; doi:10.1007/s10544-014-9847-3)
Supplement: Supplementary file 1 — (DOC 523 kb) [file 10544_2014_9847_MOESM1_ESM.doc]

Antibacterial performance of nanocrystallined titania confined in mesoporous silica nanotubes.

Krzysztof Cendrowski^±^ *, Magdalena Peruzynska^§^, Agata Markowska-Szczupak^+^, Xuecheng Chen^±^, Anna Wajda^§^, Joanna Lapczuk^§^, Mateusz Kurzawski^§^, Ryszard J. Kalenczuk^±^, Marek Drozdzik^§^, Ewa Mijowska^±^

± West Pomeranian University of Technology Szczecin, Centre of Knowledge Based Nanomaterials and Technologies,Institute of Chemical and Environment Engineering, Szczecin, Poland

^+^ West Pomeranian University of Technology Szczecin, Department of Biotechnology, Szczecin, Poland

^§^ Pomeranian Medical University, Department of Pharmacology, Szczecin, Poland

* corresponding author: [kcendrowski@zut.edu.pl](mailto:kcendrowski@zut.edu.pl) , phone number: (+48 91) 449 47 29, fax: (+48 91) 449 46 86


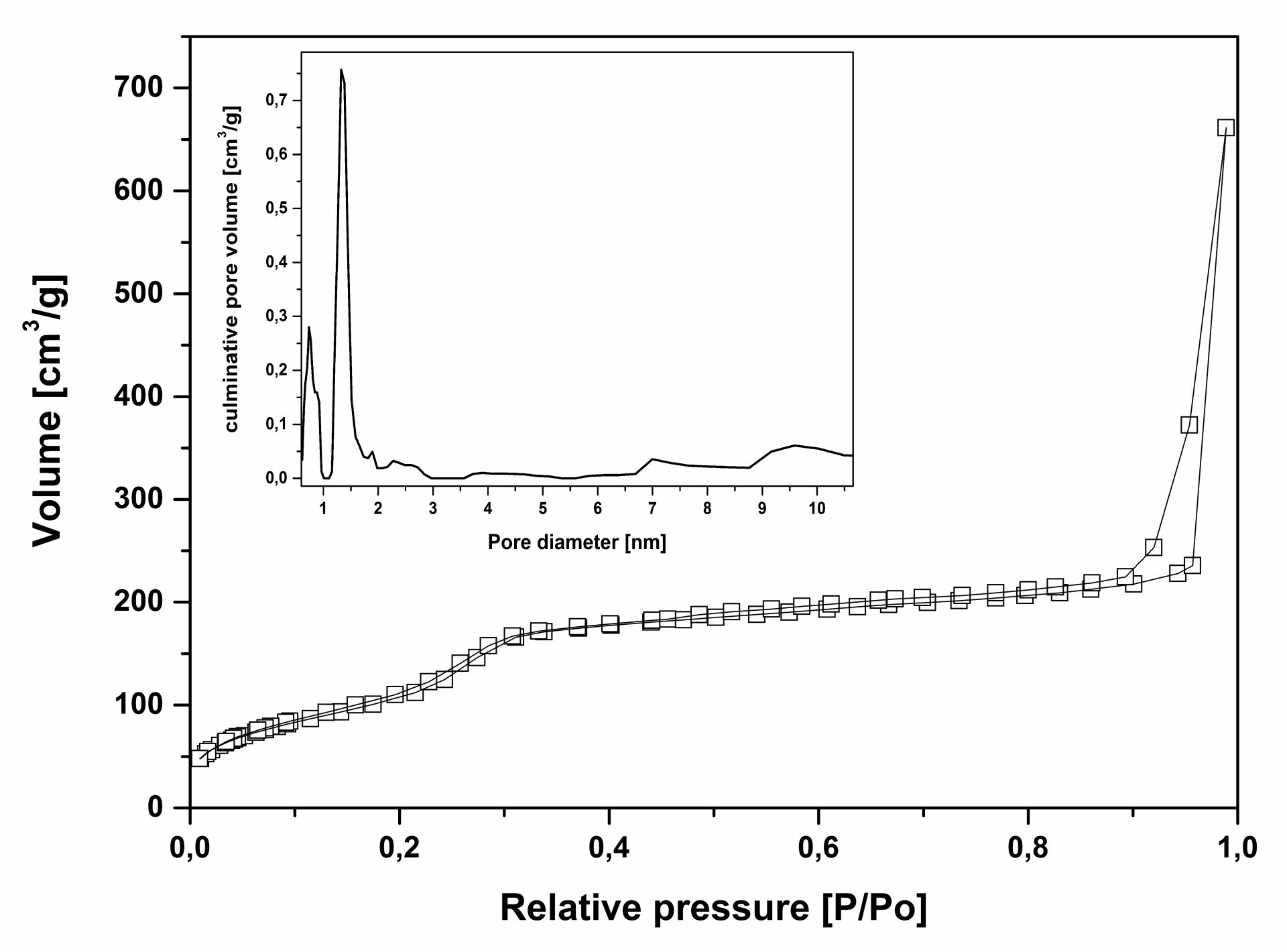


Fig. S1 N_2_ adsorption isotherms and the pore-diameter curve of the carbon nanotubes with mesoporous silica shell (CNT-mSiO_2_)


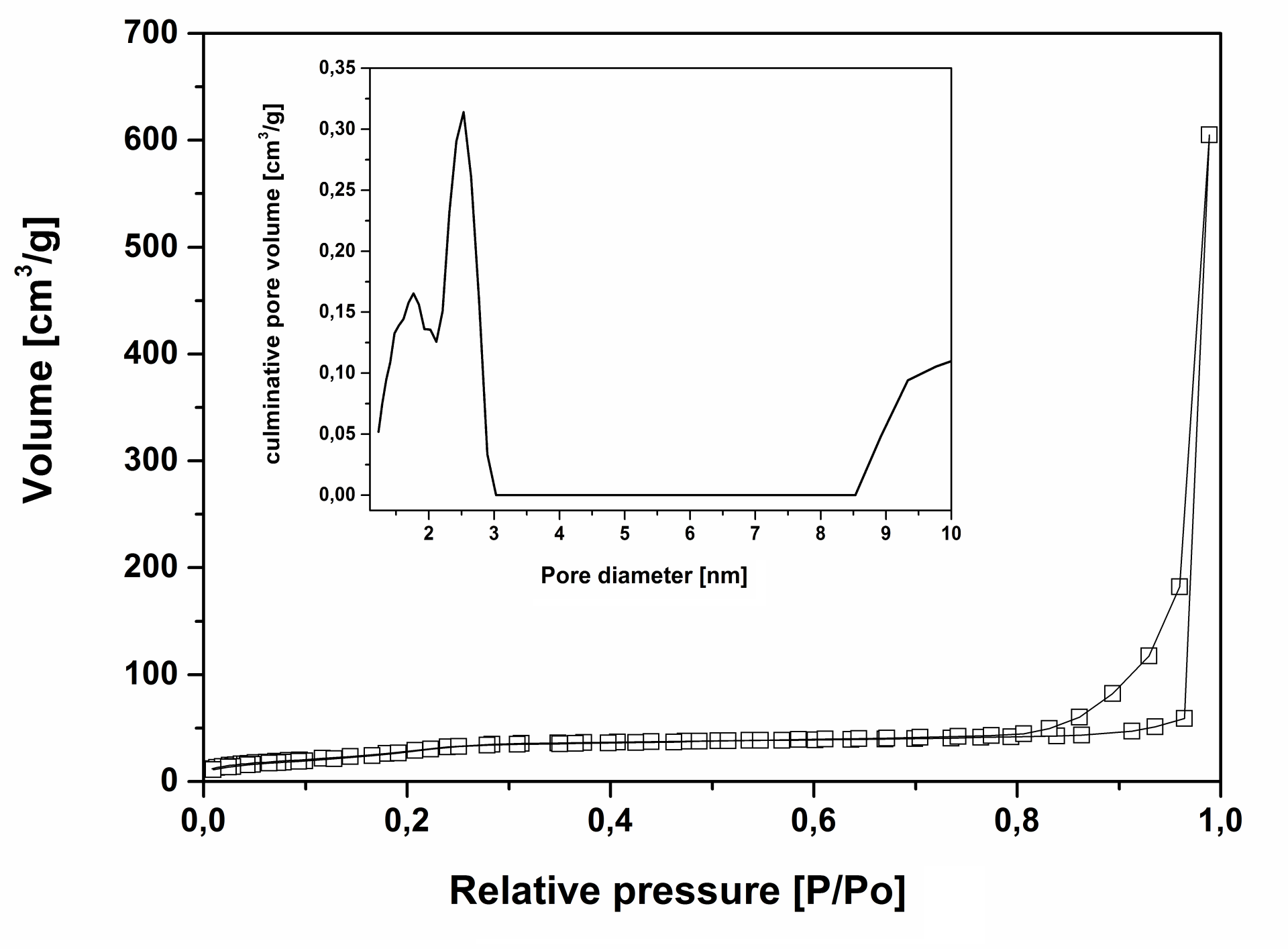


Fig. S2 N_2_ adsorption isotherms and the pore-diameter curve of the mesoporous silica nanotubes (tSiO_2_)


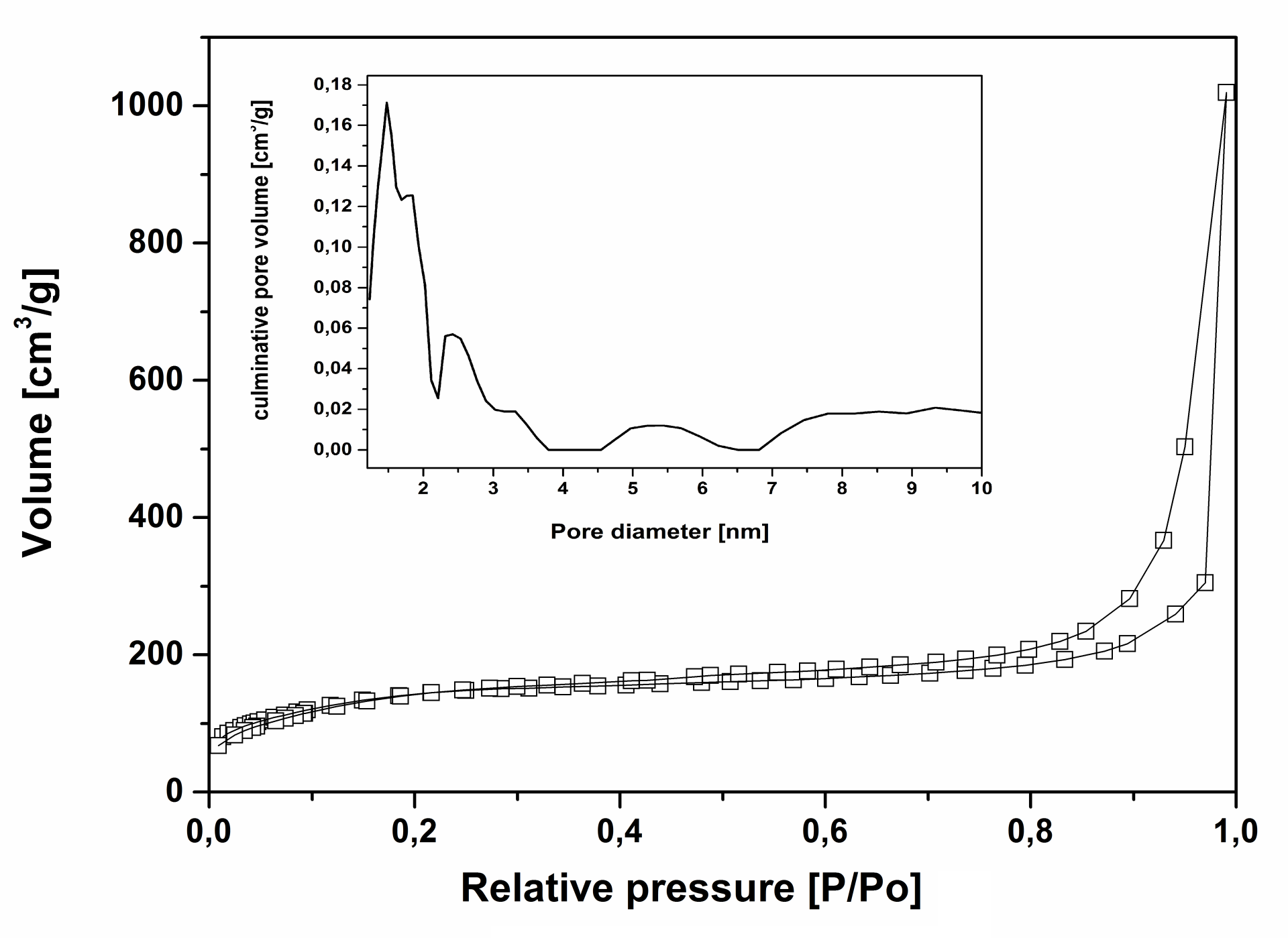


Fig. S3 N_2_ adsorption isotherms and the pore-diameter curve of the mesoporous silica nanotubes supported with titania (tSiO_2_/TiO_2_)
